# Report of a Case of Nervous Disease

**Published:** 1856-01

**Authors:** Harry Dove


*Report of a case of Nervous Disease.* By HARRY DOVE, M. R. C. S., &c.

The apparently increasing prevalence of this disease induces me to communicate a case which has come under my care.

It is that of R. K—, a clever, intelligent young woman, well known in Norwich, who was born in the year 1827. She was a very delicate baby, and continued so until two years old, at which time she was seized with brain fever, followed soon after with typhus and spotted fever; she then rallied, and was able to sit up about an hour in the day. At five years of age, it appears she was again attacked with a very severe illness, whereby she entirely lost the use of her legs, which she never regained until she was nearly twenty-three years old. At the age of six, she had so severe an attack of obstruction in the bowels, that for a fortnight she was nearly senseless from pain, and when the pain ceased lay in an almost dormant state for twelve months. From the age of nine to fourteen and a half, she had no use of her hands and arms. When ten years old, she was seized with a violent spasmodic cough, which caused her to vomit what little food she took. This continued until she recovered. At eleven her legs began to draw up. For more than sixteen years she was unable to sit up, or even to have her head raised, but lay quite flat, and unable to turn herself on a board on which she lay in the bed. She had lain in all eighteen years, during which time she was subject to severe convulsions and spasms, which lasted thirty-six and forty-eight hours at a time; she was frequently in an almost lifeless state; her knees were drawn up almost to her chin; the rib-bones seemed out of place; her foot was contracted; she was so small, that she did not measure two spans round the largest part of her body; suffered repeatedly from neuralgia of the left side, and of the face and head. She was a living skeleton, unable to feed herself, so nervous, that she could scarcely bear any one to walk in the room, and so weak that she could scarcely speak; had repeated attacks of palpitation of the heart, hiccough, yawning, and obstruction in the bowels, which caused them to protrude to a great length. During these many long and weary years she was visited by upwards of twenty physicians and surgeons, not one of whom gave her any hope of recovery.

In May, 1849, I commenced attendance in this case; she was then twenty-two years old. I found her looking pale and emaciated, with an expression of countenance which I shall never forget—an expression indicative of long-cotinued suffering. Her head was bound up, and without even a pillow; for they dared not raise her head thus much. I felt that some effort should be made to restore her, beyond the simply alleviating system which had hitherto been adopted. The treatment consisted in gradually raising the head and shoulders; in removing all her carious stumps of teeth (sixteen in number); this latter cured the neuralgia, enabled her to take more food, and her health much improved. The general impression was, that I was subjecting her to useless pain, as no good could be done. Highly *flattering* epithets were applied to me. I persisted in spite of this, and of the difficulties which beset me, and which may be easily imagined, as she had lain quite flat on her back for sixteen years and three quarters. It took three months to raise her a few inches; her limbs were gradually straightened; then depressed. At length she reached the sitting posture. She was *now* able to retain her food, and became gradually able to walk. The catamenia appeared for the first time in a year and a quarter from the commencement of the treatment. She is now in good health, capable of walking several miles, and, with the exception her being rather short, there is nothing remarkable in her personal appearance. She grew four inches after she was twenty-three years and a half old. In the following year her usual medical attendant divided the tendo-Achilles. I regret this, as I believe the contracted ankle might have been restored by other means."

The following is the condensed argument of a paper read before the Royal Medical Chirurgical Society.

"*On the Intrinsic Calcification of the Permanent Tooth-pulp as constantly associated with Dental Caries.* By S. JAMES A. SALTER, ESQ., M. B.; F. L. S.

The object of this communication was to explain the changes which take place when the dentine becomes carious, and the following are the principal points enforced:—

1. In every carious tooth the pulp becomes impregnated with calcareous matter.

2. This produces a change in its physical properties; it becomes first firmer, then natural, then hard and elastic, and perfectly hard and brittle, according to the degrees of calcification.

3. The calcification occurs intrinsically in the substance of the pulp, and not on its surface; differing in this respect from primary and the other two forms of secondary dentine.

4. The calcification displays itself at sundry isolated points, producing what the author proposes to call 'islands of calcification.'

5. The islands of calcification are not interstitial deposits of earthy matter, but consist of animal matter impregnated with earthy.

6. All the tissues of the pulp appear to sustain the impregnation, and are originally the animal basis.

7. As the calcification advances, the islands enlarge, and at length fuse together, forming a dense coherent mass; this is osteo-dentine.

8. In the perfectly calcified pulp the functions of the nerves have ceased, and the structure is insensitive.

9. Sometimes, instead of osteo-dentine, the pulp is converted into crusta petrosa.

10. The process of calcification, though morbid in its nature, is reparative in its results."

Mr. James Salter exhibited before the Pathological Society of London, a specimen of

"*Vascular cancellated tooth-bone, constituting an exostosis on the fang of the bicuspid tooth.*

The tooth, which was a superior bicuspid, was given to Mr. Salter by Mr. Walter Jones, of Worcester. The lower half of the fang was incrusted irregularly by hypertrophied tooth-bone, and exhibiting on its surface apertures and cancellated openings. A section of the tooth, viewed with low powers of the microscope, displayed many very singular vascular canals, varying in diameter from one hundredth to one thousandth of an inch. These canals run through the structure in every conceivable direction, frequently branching and varying much in diameter in different parts of their course. In all respects, this exostosis contrasts remarkably with the ordinary compact, non-vascular exostosis. The structure was illustrated by drawings and microscopical specimens."

"Mr. Salter also exhibited a specimen of

*Fatty degeneration of the tooth-pulp.*

This was observed by Mr. Salter in a tooth which had been some months removed. The pulp, which was in a soft, diffluent condition, was found, upon microscopical examination, to contain multitudes of minute, highly refracting globules, scattered amongst the *debris* of the pulp, in all respects resembling fat globules, as seen in ordinary degenerate tissues. Upon application of ether, these globules dissolved and vanished, proving their fatty nature. The dentine of this tooth was unusually clear, and of a dark yellow color; and microscopical sections were so transparent, that the tubuli could be scarcely discerned. When, however, they were boiled in ether, the white opacity of the dentine was restored, and the tubuli everywhere apparent, as in the normal tissue. This was shown in sections of the neck of the tooth."

